# Association of Socioeconomic Position With e-Cigarette Use Among Individuals Who Quit Smoking in England, 2014 to 2019

**DOI:** 10.1001/jamanetworkopen.2020.4207

**Published:** 2020-06-05

**Authors:** Loren Kock, Jamie Brown, Lion Shahab

**Affiliations:** 1 Department of Behavioural Science and Health, University College London, London, United Kingdom; 2 SPECTRUM Research Consortium, Edinburgh, United Kingdom

## Abstract

**Importance:**

e-Cigarette use among individuals who quit smoking more than 1 ago in England is highest among those with lower socioeconomic position and may affect smoking-related health inequalities, depending on whether the devices protect against relapse to tobacco smoking.

**Objectives:**

To assess trends in current e-cigarette use by socioeconomic position among individuals who have quit smoking for at least 1 year, to capture postcessation initiation among those who quit within the past year and did not use an e-cigarette in their most recent quit attempt (representing recent initiation), and to capture postcessation initiation among those who quit smoking before e-cigarettes became popular in 2011 (representing late initiation).

**Design, Setting, and Participants:**

This cross-sectional study of 34 442 adults (≥16 years) who formerly smoked used data from the Smoking Toolkit Study (conducted 2014-2019), a nationally representative, monthly, repeated, cross-sectional, household survey of smoking and smoking cessation in England. Data analysis was conducted in December 2019.

**Exposures:**

Socioeconomic position based on occupation.

**Main Outcomes and Measures:**

Current self-reported e-cigarette use.

**Results:**

Weighted samples consisted of 19 297 individuals who had quit smoking for at least 1 year (mean [SD] age, 59.2 [17.0] years; 9024 [46.8%] women), 904 who quit in the past year and did not use an e-cigarette in their most recent quit attempt (mean [SD] age, 41.6 [17.1] years; 445 [49.3%] women), and 14 241 who quit before 2011 (mean [SD] age, 63.6 [14.6] years; 6619 [46.5%] women). Among those who had quit smoking fot at least 1 year, e-cigarette use increased from 3.3% (95% CI, 2.7%-4.0%) in 2014 to 10.4% (95% CI, 9.2%-11.6%) in 2019 among all socioeconomic groups. Use was more common among those with lower socioeconomic position than those with higher socioeconomic position (odds ratio, 1.59; 95% CI, 1.05-2.40; *P* = .03). Regarding postcessation initiation of e-cigarettes, among those who quit smoking in the past year and did not use an e-cigarette in their most recent quit attempt, 7.1% (95% CI, 5.9%-9.3%) initiated e-cigarette use after smoking cessation, and there was no clear trend over time or any difference according to socioeconomic position. Among those who quit before 2011, there was an overall increase in use of e-cigarettes (0.8% [95% CI, 0.5%-1.2%] in 2014 to 2.1% [95% CI, 1.4%-2.8%] in 2019), but there were no apparent differences in use across socioeconomic position.

**Conclusions and Relevance:**

In this study, e-cigarette use increased among all participants from 2014 to 2019 but was highest among those with lower socioeconomic position. Continued monitoring of this socioeconomic patterning is important because if e-cigarettes do not confer the public health benefit of protection against relapse to smoking, then equity-negative disadvantages of long-term usage are more likely. Late, but not recent, postcessation initiation of e-cigarettes has increased over time but is not likely to affect smoking-related health inequalities because there were no differences by socioeconomic position.

## Key Points

**Question:**

Is socioeconomic position associated with use of e-cigarettes among those who formerly smoked, and has use e-cigarette use changed over time?

**Findings:**

In this cross-sectional study of 34 442 individuals who formerly smoked, e-cigarette use increased among all who had not smoked for at least 1 year but was highest among those with lower socioeconomic position. Among those who quit smoking before 2011, there was no evidence for an association between socioeconomic position and e-cigarette use.

**Meaning:**

In this study, lower socioeconomic position was associated with higher rates of e-cigarette use among those who quit smoking after e-cigarettes became widely available, likely reflecting continued use of e-cigarettes as a long-term cessation aid among individuals with lower socioeconomic positions.

## Teaser

This cross-sectional study assesses trends in current e-cigarette use by socioeconomic position among individuals who have quit smoking for at least 1 year, captures postcessation initiation among those who quit within the past year and did not use an e-cigarette in their most recent quit attempt and among those who quit smoking before 2011.

## Introduction

In most high-income countries, smoking rates are highest among groups with socioeconomic disadvantage.^1^ Considering the high rates of mortality and morbidity that smoking causes, reducing these inequalities remains an important priority.

e-Cigarettes are the most popular support for quitting smoking in England,^2^ with increasing evidence for the devices as an effective method for smoking cessation.^3,4^ They are generally thought to confer substantially lower levels of harm to users compared with combusted tobacco.^5,6,7,8^ Until evidence emerges regarding the devices’ protection against relapse to smoking in the longer term, it is important to monitor the increasing number of individuals who formerly smoked^9^ and now use e-cigarettes^2^ because without the benefit of protecting against relapse to smoking, their long-term use is likely to be harmful.

There are several possible trajectories of use among individuals who formerly smoked. Users may initiate e-cigarette use before or during a quit attempt and continue to use them in the long term once they have quit.^4,10^ Alternatively, as will be assessed in this study, e-cigarette initiation may occur among individuals who formerly smoked but did not use an e-cigarette in their quit attempt. This initiation may occur within a year of quitting or in the longer term.

US research using National Health Interview Survey data indicated that individuals with higher education were more likely to transition from smoking to exclusive e-cigarette use compared with individuals with less educational attainment.^11^ Also in the US, recent Population Assessment of Tobacco and Health Study data have shown that exclusive e-cigarette use was more likely among higher-income smokers.^12^ These findings contrast with the current evidence on e-cigarette use in the UK. In England specifically, there are no apparent differences in e-cigarette use during a quit attempt between smokers by socioeconomic position, but a social gradient in use by those who quit smoking for at least 1 year exists, such that there appears to be greater use among groups with lower socioeconomic position.^13,14^ Therefore, it is likely that individuals who formerly smoked with higher socioeconomic position are using e-cigarettes during a quit attempt before discontinuing their use. In contrast, a greater proportion of individuals who formerly smoked with lower socioeconomic position may continue to use e-cigarettes after their smoking quit attempt or to initiate e-cigarettes after smoking cessation. However, these potential socioeconomic position trajectories have not been examined in detail. Individuals who smoke and have socioeconomic disadvantage are thought to be more dependent on nicotine because of generally initiating smoking at a younger age and smoking more cigarettes per day.^15^ Such dependence might encourage greater use of e-cigarettes after quitting for pleasure, to satisfy cravings, and potentially to prevent relapse to smoking.^16,17^

e-Cigarettes have been on the UK market for approximately a decade, allowing for an examination of the trends in use among individuals who formerly smoked and are using the devices. Using data from the Smoking Toolkit Study (2014-2019), this study’s aims were to assess trends in e-cigarette use according to socioeconomic position by examining current use of e-cigarettes among all individuals who had quit smoking for at least 1 year and recent and late initiation of e-cigarettes after smoking cessation, respectively, among those who quit smoking in the past year but did not use an e-cigarette in their quit attempt (recent initiation) and those quit smoking before e-cigarettes became popular in 2011 (late initiation). To contextualize observed trends of e-cigarette use against another widely available nicotine delivery product, this study also assessed trends in the use of nicotine replacement therapy by socioeconomic position in the same categories listed earlier.

## Methods

### Study Design

This study followed a repeated cross-sectional survey design. Smoking Toolkit Study data collected from January 2014 to September 2019 were used. We selected 2014 as the referent year because it was the first year in which the Smoking Toolkit Study measured e-cigarette use among all respondents.

The analytic sample consisted of adults aged 16 years and older living in households in England. The Smoking Toolkit Study involves monthly cross-sectional household interviews of 1700 to 1800 adults, conducted by the market research specialist Ipsos MORI. Sampling of participants for the baseline survey uses a hybrid of random probability and simple quota sampling. This involves assorting England into more than 170 000 initial output areas made up of approximately 300 households, stratified by the 9 regions of England. Interviews are then conducted with a single member of households within randomly assigned stratified output areas. This continues in each area until quotas based on area demographic characteristics are fulfilled. Given the high number of output areas included in each wave, which are themselves randomly sampled from more than 170 000 initial output areas, it is unlikely that there are substantial clusters resulting in bias. All cases were weighted with the rim (marginal) weighting technique to match the English population profile relevant to the time each monthly survey was collected.^18^

The Smoking Toolkit Study was approved by University College London’s research ethics committee, and participants provided full written informed consent. The data are anonymized when received by University College London. The Strengthening the Reporting of Observational Studies in Epidemiology (STROBE) reporting guideline were used in the design and reporting of this study.^19^

### Measures

#### Main Outcomes

##### e-Cigarette and Nicotine Replacement Therapy Use Among Individuals Who Quit Smoking For at Least 1 Year

Responses to the question “smoking status?” were used to identify those who had quit smoking for at least 1 year. Responses to the question, “Can I check whether you are using any of the following?” were used to identify the nicotine product being used. Answer selections of e-cigarette and nicotine replacement therapy (which included nicotine patch, nicotine gum, nicotine lozenges or tablets, nicotine inhaler, and nicotine nasal spray) in response to the question were used as the outcome measures for e-cigarette and nicotine replacement therapy usage, respectively, among respondents.

##### Recent Initiation of e-Cigarette or Nicotine Replacement Therapy Among Those Who Quit Smoking in the Past Year But Did Not Use These Products in Their Most Recent Quit Attempt

Responses to the question “What did you use to help stop smoking during the most recent serious quit attempt?” were used to identify those who quit smoking in the past year and used either an e-cigarette or nicotine replacement therapy in their quit attempt. Answer selection of e-cigarette or any form of nicotine replacement therapy indicated respective product use and triggered exclusion of such participants from the subsequent analysis. Current e-cigarette or nicotine replacement therapy use was then ascertained with answers of e-cigarette and nicotine replacement therapy, respectively, in response to the question “Can I check whether you are using any of the following?” Therefore, the remaining sample included those who quit smoking within the past year and who started using an e-cigarette or nicotine replacement therapy only after they had quit smoking. Prevalence of use of e-cigarettes and nicotine replacement therapy among this group was calculated by counting the number of respondents who endorsed use of either product to the previously mentioned question outside of a quit attempt, divided by the number of individuals who reported quitting without assistance from the respective products.

##### Late Initiation of e-Cigarettes Among Individuals Who Quit Smoking Before 2011

As a measure of late initiation of e-cigarette use, we assessed current e-cigarette use among those who had quit before e-cigarettes became popular in 2011 (ie, >8 years ago). Length of abstinence (how many years ago a respondent quit smoking) was calculated as actual age minus the age when the respondent stopped smoking.

To create subgroups of respondents for each year from 2014 to 2019 who had quit smoking before 2011, the length of abstinence calculated as mentioned earlier was used as follows: 2014, respondents with length of abstinence at least 4 years; 2015, those with length of abstinence at least 5 years; 2016, those with length of abstinence at least 6 years; 2017, those with length of abstinence at least 7 years; 2018, those with length of abstinence at least 8 years; and 2019, those with length of abstinence at least 9 years. Current e-cigarette use was ascertained as described earlier in response to the question, “Can I check whether you are using any of the following?” Answer selections of electronic cigarette in response to the question were used as the outcome measures for e-cigarette usage. Prevalence of e-cigarette use among this group was calculated by counting the number of respondents who endorsed use of e-cigarettes, divided by the total number of individuals who quit smoking before 2011.

### Socioeconomic Variables

Smoking Toolkit Study respondents were stratified according to socioeconomic position with the National Readership Survey classification system for social grade based on occupation.^20^ This measure typically contains 5 categories (ie, AB, C1, C2, D, and E) but was collapsed to 2 groups with higher and intermediate managerial, administrative and professional, supervisory, clerical, and junior managerial occupations operationalized as a proxy for higher socioeconomic position; and semiskilled and unskilled manual workers, state pensioners, casual and lowest-grade workers, and unemployed with state benefits only operationalized as a proxy for lower socioeconomic position.

Living in social housing has been reported to be an independent risk factor for smoking in England.^21^ Therefore, sensitivity analyses were conducted with housing tenure^22^ as an alternative measure of socioeconomic position. This measure was collapsed to 2 groups, social housing (local authority or housing association) and other (mortgage bought, owned outright, private renting, and other).

### Covariates

#### Adjusting e-Cigarette and Nicotine Replacement Therapy Use for Background Popularity of Each Respective Product

Prevalence of e-cigarettes and nicotine replacement therapy use was adjusted for background popularity of each product in the general population. We replaced the denominator for each subgroup with the general population denominator and expressed the prevalence as a percentage of use in the general population.

#### Sociodemographic Characteristics

The sociodemographic characteristics sex (categorized as women or other) and age (categories 16-24, 25-34, 35-44, 45-54, 55-64, and ≥65 years) were measured. We also measured region in England (London, North East, North West, Yorkshire and Humber, East Midlands, West Midlands, East of England, South East, and South West).

### Statistical Analysis

To assess trends in the associations between e-cigarette or nicotine replacement therapy use and year among ex-smokers, we constructed multivariable logistic regression models including survey year (categoric predictor variable with referent year 2014) and socioeconomic position (2 categories with the following referent: higher and intermediate managerial, administrative and professional, supervisory, clerical, and junior managerial occupations) and the interaction terms. All models were adjusted for age, sex, and region. Data analysis was carried out in R version 3.5.2 (R Project for Statistical Computing) in December 2019. A 2-sided *P* < .05 was considered statistically significant. The analysis plan was preregistered on the Open Science Framework. Participants with missing data for any of the variables in the analyses were excluded.

All associations are reported stratified by year. Odds ratios (ORs) with 95% CIs (adjusted for age, sex, and region) indicate the associations between e-cigarette use and year or socioeconomic position. The inclusion of the socioeconomic position–by-year interaction terms allowed us to examine any differential time trends according to socioeconomic position.

Sensitivity analyses were run with housing tenure as an alternative measure of socioeconomic position. Results on prevalence of use for the groups described earlier were also plotted with adjustment for background popularity in the general population.

Quasi-complete separation occurred in the yearly stratified models involving individuals who quit smoking in the last year. To resolve this, the following covariates were merged to become dichotomous predictors (age, 16-44 vs ≥45 years; region, south vs north).

## Results

A weighted total of 19 297 individuals who quit smoking for more than 1 year (mean [SD] age, 59.2 [17.0] years; 9024 [46.8%] women), 904 who quit in the past year and did not use an e-cigarette in their recent quit attempt (mean [SD] age 41.6 [17.1] years; 445 [49.3%] women), and 1353 who quit in the past year and did not use nicotine replacement therapy in their recent quit attempt (mean [SD] age, 40.6 [16.6]; 631 [46.6%] women) in England completed the baseline Smoking Toolkit Study survey between January 2014 and September 2019. The subgroup of individuals (2014-2019) who had quit before 2011 included 14 241 respondents (mean [SD] age, 63.6 [14.6] years; 6619 [46.5%] women) (Table 1).

**Table 1.** Characteristics of Sample, Weighted Data

Variables	Participants, No. (%)			
	Quit >1 y (n = 19 297)^a^	Quit before 2011 (n = 14 241)^b^	Quit <1 y (n = 904)^c^	Quit <1 y (n = 1353)^d^
e-Cigarette use				
Yes	1361 (7.05)	155 (1.09)	67 (7.41)	NA
No	17 936 (92.95)	14 086 (98.91)	837 (92.59)	NA
NRT use				
Yes	590 (3.06)	317 (2.23)	NA	50 (3.69)
No	18 707 (96.94)	13 923 (97.77)	NA	1303 (96.31)
Sex				
Women	9024 (46.77)	6619 (46.48)	445 (49.26)	631 (46.59)
Age, y				
16-24	521 (2.70)	37 (0.26)	153 (16.87)	241 (17.84)
25-34	1814 (9.40)	596 (4.18)	241 (26.61)	340 (25.13)
35-44	2571 (14.26)	1707 (11.99)	193 (21.36)	300 (22.17)
45-54	3434 (17.80)	2463 (17.30)	143 (15.80)	228 (16.82)
55-64	3555 (18.42)	2885 (20.26)	92 (10.13)	139 (10.24)
≥65	7222 (37.42)	6552 (46.01)	83 (9.23)	106 (7.81)
SEP				
ABC1	11 408 (59.12)	8776 (61.63)	478 (52.89)	693 (51.22)
C2DE	7889 (40.88)	5464 (38.37)	426 (47.11)	660 (48.78)

Abbreviations: ABC1, higher and intermediate managerial, administrative and professional, supervisory occupations, and clerical and junior managerial occupations; C2DE, semiskilled and unskilled manual workers, state pensioners, casual and lowest-grade workers, and unemployed with state benefits only; NA, not applicable; NRT, nicotine replacement therapy; SEP, socioeconomic position.

Ns are weighted. ABC1 indicates a higher SEP; C2DE, a lower one.

^a^ All participants who quit smoking for at least 1 year.

^b^ Individuals who quit smoking before 2011.

^c^ Individuals who quit smoking in the past year and did not use e-cigarettes in their most recent quit attempt.

^d^ Individuals who quit smoking in the past year and did not use NRT in their most recent quit attempt.

### e-Cigarette Use Among Those Who Quit Smoking for More Than 1 Year

e-Cigarette use among those who quit more than 1 year ago increased from 3.30% (95% CI, 2.72%-3.96%) in 2014 to 10.36% (95% CI, 9.19%-11.62%) in 2019 among all socioeconomic groups. All respondents, regardless of socioeconomic position, were more likely to use e-cigarettes in each year from 2016 to 2019 compared with 2014 (eg, 2017: OR, 2.88; 95% CI, 2.06-4.09; *P* < .001; 2018: OR, 3.19; 95% CI, 2.29-4.54; *P* < .001; 2019: OR, 3.46; 95% CI, 2.45-4.97; *P* < .001) (Figure, A and Table 2). There were no significant interactions between socioeconomic position and time. Overall, respondents with lower socioeconomic position were more likely to use e-cigarettes compared with more affluent respondents (OR, 1.59; 95% CI, 1.05-2.40; *P* = .03). When stratified by year, this socioeconomic gradient in e-cigarette use remained, with the exception of 2016, when there was no clear evidence of an association with socioeconomic position (eg, 2017: OR, 1.33; 95% CI, 1.03-1.73; *P* = .03; 2018: OR, 1.35; 95% CI, 1.05-1.74; *P* = .02; 2019: OR, 1.59; 95% CI, 1.19-2.11; *P* = .001) (Table 3).

**Figure.**
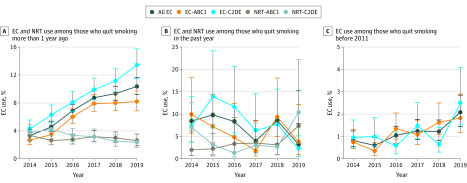
Trends in e-Cigarette (EC) and Nicotine Replacement Therapy (NRT) Use Among Individuals Who Quit Smoking in England, 2014-2019 ABC1 indicates higher and intermediate managerial, administrative and professional, supervisory occupations, and clerical and junior managerial occupations; and C2DE, semiskilled and unskilled manual workers, state pensioners, casual and lowest-grade workers, and unemployed with state benefits only. Whiskers indicate 95% CIs.

**Table 2.** Associations Between e-Cigarette Use and Year of Survey and Socioeconomic Position^a^

Variable	Quit >1 y (n = 19 842)^b^		Quit <1 y (n = 870)^c^		Quit before 2011 (n = 15 063)^d^	
	OR (95% CI)	*P* value	OR (95% CI)	*P* value	OR (95% CI)	*P* value
SEP						
ABC1	1 [Reference]		1 [Reference]		1 [Reference]	
C2DE	1.59 (1.05-2.40)	.03	0.54 (0.18-1.55)	.26	1.25 (0.47-3.21)	.62
Year						
2014	1 [Reference]		1 [Reference]		1 [Reference]	
2015	1.29 (0.88-1.90)	.20	0.54 (0.16-1.67)	.31	0.43 (0.13-1.22)	.13
2016	2.34 (1.66-3.37)	<.001	0.42 (0.09-1.52)	.21	1.93 (0.92-4.32)	.09
2017	2.88 (2.06-4.09)	<.001	0.18 (0.03-0.73)	.03	1.50 (0.68-3.47)	.32
2018	3.19 (2.29-4.54)	<.001	0.70 (0.22-2.10)	.54	2.79 (1.37-6.13)	.006
2019	3.46 (2.45-4.97)	<.001	0.31 (0.05-1.28)	.22	3.29 (1.56-7.40)	.005
Interaction terms						
2015 × C2DE	1.23 (0.73-2.10)	.44	4.63 (0.95-24.13)	.07	2.39 (0.56-10.9)	.25
2016 × C2DE	0.79 (0.48-1.31)	.36	4.53 (0.81-29.90)	.09	0.47 (0.12-1.72)	.26
2017 × C2DE	0.85 (0.52-1.37)	.50	7.21 (1.06-65.99)	.050	1.35 (0.41-4.57)	.63
2018 × C2DE	0.87 (0.54-1.40)	.57	1.39 (0.24-7.63)	.72	0.39 (0.11-1.37)	.15
2019 × C2DE	0.97 (0.59-1.60)	.67	1.70 (0.07-23.82)	.95	0.99 (0.30-3.31)	.98

Abbreviations: ABC1, higher and intermediate managerial, administrative and professional, supervisory occupations, and clerical and junior managerial occupations; C2DE, semiskilled and unskilled manual workers, state pensioners, casual and lowest-grade workers, and unemployed with state benefits only; LT, longterm; OR, odds ratio; PY, pastyear; SEP, socioeconomic position.

^a^ Numbers are not weighted. All models are adjusted for age, sex, and region. Results for prevalence of e-cigarette use are presented as ORs (95% CI) against the indicated referent.

^b^ All participants who quit smoking for at least 1 year.

^c^ Individuals who quit smoking in the past year and did not use e-cigarettes in their most recent quit attempt.

^d^ Individuals who quit smoking before 2011.

**Table 3.** Associations Between e-Cigarette Use and Socioeconomic Position Stratified by Year

SEP	2014		2015		2016		2017		2018		2019	
	OR (95% CI)	*P* value	OR (95% CI)	*P* value	OR (95% CI)	*P* value	OR (95% CI)	*P* value	OR (95% CI)	*P* value	OR (95% CI)	*P* value
**Quit >1 y**^**a**^												
No.	3170	NA	3462	NA	3533	NA	3617	NA	3532	NA	2528	NA
ABC1 (n = 12 008)	1 [Reference]	NA	1 [Reference]	NA	1 [Reference]	NA	1 [Reference]	NA	1 [Reference]	NA	1 [Reference]	NA
C2DE (n = 7834)	1.64 (1.08-2.5)	.02	2.01 (1.43-2.82)	<.001	1.27 (0.95-1.7)	.10	1.33 (1.03-1.73)	.03	1.35 (1.05-1.74)	.02	1.59 (1.19-2.11)	.001
**Quit <1 y**^**b**^												
No.	194	NA	158	NA	129	NA	152	NA	152	NA	85	NA
ABC1 (n = 479)	1 [Reference]	NA	1 [Reference]	NA	1 [Reference]	NA	1 [Reference]	NA	1 [Reference]	NA	1 [Reference]	NA
C2DE (n = 391)	0.61 (0.21-1.73)	.35	3.46 (1.05-12.64)	.04	2.24 (0.56-11.18)	.27	3.12 (0.61-23.06)	.20	0.80 (0.19-3.07)	.75	0.97 (0.04-11.07)	.63
**Quit before 2011**^**c**^												
No.	2683	NA	2805	NA	2703	NA	2649	NA	2501	NA	1722	NA
ABC1 (n = 9394)	1 [Reference]	NA	1 [Reference]	NA	1 [Reference]	NA	1 [Reference]	NA	1 [Reference]	NA	1 [Reference]	NA
C2DE (n = 5669)	1.12 (0.42-2.87)	.81	3.22 (1.06-10.81)	.04	0.51 (0.18- 1.21)	.15	1.74 (0.81-3.74)	.15	0.45 (0.18-1.00)	.06	1.20 (0.56-2.47)	.52

Abbreviations: ABC1, higher and intermediate managerial, administrative and professional, supervisory occupations, and clerical and junior managerial occupations; C2DE, semiskilled and unskilled manual workers, state pensioners, casual and lowest-grade workers, and unemployed with state benefits only; NA, not applicable; OR, odds ratio; SEP, socioeconomic position.

Ns are not weighted. All models are adjusted for age, sex, and region. Results for prevalence of e-cigarette use are presented as ORs (95% CI) against the indicated referent. ABC1 indicates a higher SEP; C2DE, a lower one.

^a^ All participants who quit smoking for at least 1 year.

^b^ Individuals who quit smoking in the past year and did not use e-cigarettes in their most recent quit attempt.

^c^ Individuals who quit smoking before 2011.

There was no clear trend in nicotine replacement therapy use across time or association between socioeconomic position and nicotine replacement therapy use, with the exception of 2015, in which individuals with lower socioeconomic position who had quit smoking for at least 1 year were more likely to use nicotine replacement therapy compared with those in the higher socioeconomic position group (OR, 1.66; 95% CI, 1.11-2.48; *P* = .02) (eTable 1 in the Supplement).

As a percentage of use in the general population, e-cigarette use increased from 10.53% (95% CI, 8.76%-12.60%) in 2014 to 30.48% (95% CI, 27.43%-33.72%) in 2019. This trend was also evident across socioeconomic position groups, increasing from 11.30% (95% CI, 8.73%-14.52%) to 31.88% (95% CI, 27.32%-36.81%) in the higher socioeconomic position group (higher and intermediate managerial, administrative and professional, supervisory, clerical, and junior managerial occupations) and 9.84% (95% CI, 7.59%-12.69%) to 29.36% (95% CI, 25.35%-33.71%) in the lower socioeconomic position group (semiskilled and unskilled manual workers, state pensioners, casual and lowest-grade workers, and unemployed with state benefits only) (eFigure 1 in the Supplement).

### Recent Initiation of e-Cigarettes After Smoking Cessation in the Past Year

With the exception of 2017, in which e-cigarette use was less likely compared with 2014, there was no clear trend over time for e-cigarette use (Figure, B and Table 2) or nicotine replacement therapy use (eTable 1A in the Supplement) in this subgroup. With the exception of 2015, in which use was more likely among respondents with lower socioeconomic position, there were no apparent differences according to socioeconomic position in each year (OR, 3.46; 95% CI, 1.05-12.64; *P* = .04) (Table 3). There was little interaction between socioeconomic position and time. The exception was that use in the lower socioeconomic position group (semiskilled and unskilled manual workers, state pensioners, casual and lowest-grade workers, and unemployed with state benefits only) depended on year, with higher comparative prevalence in 2017 compared with 2014 (OR, 7.21; 95% CI, 1.06-66.0; *P* = .050).

Overall, e-cigarette use as a percentage of use in the general population decreased from 1.72% (95% CI, 1.07%-2.74%) in 2014 to 0.37% (95% CI, 0.12%-1.07%) in 2019 (eFigure 2 in the Supplement). In the higher socioeconomic position group, use decreased from 2.17% (95% CI, 1.19%-3.95%) in 2014 to 0.54% (95% CI, 0.15%-1.96%) in 2019. In the lower socioeconomic position group, use decreased from 1.5% (95% CI, 0.77%-2.96%) in 2014 to 0.22% (95% CI, 0.04%-1.24%) in 2019.

### Late Initiation of e-Cigarettes Among Individuals Who Quit Smoking Before 2011

A trend in e-cigarette use across time was evident, whereby all respondents, regardless of socioeconomic position, were more likely to use e-cigarettes in 2018 (OR, 2.79; 95% CI, 1.37-6.13; *P* = .006) and 2019 (OR, 3.29; 95% CI, 1.56-7.40; *P* = .005) compared with 2014 (Figure, C and Table 2). There were no interactions between socioeconomic position and time. With the exception of 2015, in which use was more likely among respondents with lower socioeconomic position (OR, 3.22; 95% CI, 1.06-10.81; *P* = .04) , there were no apparent differences according to socioeconomic position in each year (Table 3).

Overall e-cigarette use among those who quit smoking before 2011 as a percentage of use in the general population was estimated to have increased from 2.23% (95% CI, 1.47%-3.35%) in 2014 to 4.02% (95% CI, 2.88%-5.60%) in 2019 (eFigure 3 in the Supplement). In the higher socioeconomic position group, use increased from 2.82% (95% CI, 1.66%-4.77%) in 2014 to 4.90% (95% CI, 3.12%-7.62%) in 2019. In the lower socioeconomic position group, use increased from 1.70% (95% CI, 0.90%-3.21%) in 2014 to 3.31% (95% CI, 2.02%-5.39%) in 2019.

### Sensitivity Analyses

In a sensitivity analysis, we used housing tenure as an alternative measure of socioeconomic position. This yielded a pattern of results similar to that of the main analysis (eTables 2 and eTable 3 in the Supplement).

## Discussion

From 2014 to 2019 in England, e-cigarette use increased among individuals who had quit smoking for more than 1 year and was highest among respondents with lower socioeconomic position. Among those who quit smoking in the past year and did not use an e-cigarette during their most recent quit attempt, there were no clear trends over time or socioeconomic differences in e-cigarette use. Use among those who quit smoking before 2011 was greater in 2018 and 2019 compared with 2014, but there was no clear evidence of a difference according to socioeconomic position throughout the period.

The findings on e-cigarette use among those who quit smoking for more than 1 year reveal a continuation of the trends observed in previous Smoking Toolkit Study data from England,^13^ with overall increased use over time and greater use among respondents with lower socioeconomic position compared with those with higher socioeconomic position. This is concordant with recently published data from the UK Household Longitudinal Study, in which socioeconomic disadvantage was associated with e-cigarette use among adults who formerly smoked.^14^ However, this recent study included respondents who self-reported as currently, formerly, or never smoked and could not explore e-cigarette use in specific subcategories of these groups, as we have attempted to do in this study.

Findings from the US population, in which e-cigarettes are similarly popular, appear to show a divergent trend, whereby e-cigarette use is more likely among individuals with socioeconomic advantage who used to smoke.^11,12^ This heterogeneity in e-cigarette use by socioeconomic position between different countries was highlighted by a 2019 systematic review in which analysis of studies from 11 countries found mixed patterns of e-cigarette current use by socioeconomic position.^23^ Such differences may be a reflection of different health policy and advocacy environments toward e-cigarettes in England compared with other countries in which the devices have become popular. However, these comparisons are made with caution; more granular data on e-cigarette use by smoking status are required from other contexts before direct comparisons with the UK can be made.

In this study, individuals who had quit smoking for more than 1 year were also found to make up an increasing percentage of e-cigarette users in the general population. These results likely reflect the continued use of e-cigarettes after smoking cessation. Comparing the trends in use by socioeconomic position highlights that although there have been consistent increases among those who quit smoking for more than 1 year with lower socioeconomic position, this is not apparent among respondents with higher socioeconomic position, whose use appears to have stabilized. Such socioeconomic patterning in e-cigarette use is important, considering that studies have shown that those who formerly smoked and now vape reported greater confidence in not smoking compared with those who do not vape.^16,24^ This is contextualized by the absence of a trend in the use of nicotine replacement therapy, another widely available nicotine product, across the period. However, a 2019 randomized clinical trial that showed e-cigarettes to be almost twice as effective for smoking cessation at 1 year compared with nicotine replacement therapy^3^ has indicated that, in the context of the trial, the devices may not confer extra protection against relapse. The trial found that time to relapse and relapse rates among participants with sustained abstinence at 4 weeks were similar between the 2 trial groups despite large differences in ongoing use (80% of individuals in the e-cigarette arm were using their allocated product at 52 weeks compared with 9% in the nicotine replacement therapy arm). Nicotine replacement therapy is likely more widely used as a medium-term quitting aid, unlike e-cigarettes, which appear more popular for longer-duration use. Therefore, compared with nicotine replacement therapy, the added value of e-cigarettes may not be because of greater protection against relapse, but rather because of greater efficacy for smoking cessation.^25^

The current absence of evidence on relapse protection along with increasing levels of prolonged use among individuals with lower socioeconomic position who formerly smoked make this area an urgent research priority. If e-cigarettes do not confer the public health benefit of protection against relapse to smoking, then equity-negative outcomes of long-term usage are more likely because although safer than smoking, use of the devices is not without risk.

The use of e-cigarettes among individuals who quit smoking in the past year and did not use an e-cigarette in their most recent quit attempt did not appear to change over time or differ by socioeconomic position. Therefore, any potentially positive or negative outcomes from the use of e-cigarettes at the population level will likely affect people equally across the socioeconomic spectrum, making it unlikely that use in this specific subgroup will have a substantial effect on smoking-related health inequalities.

The use of e-cigarettes among individuals who quit smoking before 2011 increased over time overall and was significantly higher toward the end of the period compared with 2014. This subgroup also made up an increasing percentage of e-cigarette users in the general population. Although the change appears modest, it suggests that in addition to continued e-cigarette use as a quitting aid, increases in their use may in part be due to late initiation among individuals who have already quit smoking. Those who quit smoking before 2011 represent those who quit smoking before e-cigarettes arrived on the UK market. This older (mean age, 63.6 years) subgroup may have taken up e-cigarettes to prevent relapse to tobacco smoking or because of the attractive nicotine delivery properties and decreased harm profile of the devices. Some researchers may view the latter as relapse to nicotine dependence. Given that these individuals had maintained abstinence from smoking without any assistance from e-cigarettes, it is plausible that they would have been able to remain abstinent in their absence. Our findings suggest that the initiation of e-cigarettes in this group is unlikely to affect health inequalities, but further research is needed to understand the reasons behind the observed increase in use overall. Recent prospective research in the US has suggested that use of e-cigarettes in this subgroup may increase the likelihood of a return to tobacco smoking.^26^ If the devices were increasing the likelihood of a return to smoking in England, then we would expect the prevalence of e-cigarette use among this group to stabilize or decline as many of them return to smoking. This is unlikely according to evidence from this study. Furthermore, we would also expect to observe a decrease in the rate of reduction of smoking after the advent of e-cigarettes. This does not appear to be the case, with consistent reductions in smoking year after year.^2^ Respondents in the sample of individuals who quit smoking some time ago in the aforementioned US study who reported relapsing to combustible tobacco smoking in the past 30 days were more likely to be younger (18-34 vs ≥35 years). This may represent a phenomenon unique to the US context, in which, although strongly associated with lifetime tobacco use and perhaps a reflection of common liability to addiction, youth e-cigarette use has sharply increased in recent years.^27,28,29^ This is in contrast to the United Kingdom, in which weekly e-cigarette use among former smoking youths (11-18 years) is estimated to be 3.3%^30^ and almost negligible among those who never smoked.

### Strengths and Limitations

Strengths of this study include its large representative sample of the population in England, allowing a detailed and generalizable interrogation of e-cigarette use over time in specific subgroups of individuals who formerly smoked. Furthermore, the use of a different measure of socioeconomic position in a sensitivity analysis provided results convergent with those of the main analysis. However, the results are limited by the use of self-reported cross-sectional survey data, in which smoking status was not biochemically verified. It is also challenging to measure e-cigarette consumption levels accurately without a validated quantifiable measure.

## Conclusions

This study found that from 2014 to 2019 in England, e-cigarette use increased among all individuals who had quit smoking for more than 1 year and was most likely among respondents with lower socioeconomic position. Use among those who quit in the past year and did not use an e-cigarette in their most recent quit attempt was similar over time and by socioeconomic position. Among individuals who quit smoking before 2011, e-cigarette use has increased since 2014, but there were no differences according to socioeconomic position. Therefore, this socioeconomically patterned use of e-cigarettes is unlikely because of recent or late initiation of e-cigarettes after quitting and probably reflects continued use of e-cigarettes as a long-term cessation aid in individuals of lower socioeconomic position.

## Notes

Supplement. **eTable 1. **Associations Between Nicotine Replacement Therapy, Year, and Socioeconomic Position **eTable 2. **Associations Between e-Cigarette Use and Year of Survey and SEP (Housing Tenure) in England, 2014 to 2019 **eTable 3. **Associations Between e-Cigarette Use and SEP (Housing Tenure) Stratified by Year in England, 2014-2019 **eFigure 1. **EC and NRT Use Among Those Who Quit Smoking for More Than 1 Year as a Percentage of Respective Use in the General Population **eFigure 2. **EC Use Among Those Who Quit Smoking in the Past Year and Did not Use ECs in Quit Attempt as a Percentage of Respective Use in the General Population **eFigure 3. **Current e-Cigarette Use Among Those Who Quit Smoking Before 2011 as a Percentage of Use in the General Population
