# Supplementary material for: Association of Socioeconomic Position With e-Cigarette Use Among Individuals Who Quit Smoking in England, 2014 to 2019
Source: JAMA Netw Open. 2020 Jun 5;3(6):e204207. doi: 10.1001/jamanetworkopen.2020.4207 (PMC7275246; doi:10.1001/jamanetworkopen.2020.4207)
Supplement: Supplement. — eTable 1. Associations Between Nicotine Replacement Therapy, Year, and Socioeconomic Position eTable 2. Associations Between e-Cigarette Use and Year of Survey and SEP (Housing Tenure) in England, 2014 to 2019 eTable 3. Associations Between e-Cigarette Use and SEP (Housing Tenure) Stratified by Year in England, 2014-2019 eFigure 1. EC and NRT Use Among Those Who Quit Smoking for More Than 1 Year as a Percentage of Respective Use in the General Population eFigure 2. EC Use Among Those Who Quit Smoking in the Past Year and Did not Use ECs in Quit Attempt as a Percentage of Respective Use in the General Population eFigure 3. Current e-Cigarette Use Among Those Who Quit Smoking Before 2011 as a Percentage of Use in the General Population [file jamanetwopen-3-e204207-s001.pdf]

Supplementary Online Content

Kock L, Brown J, Shahab L. Association of socioeconomic position with e-cigarette use among individuals who quit smoking in England, 2014 to 2019. *JAMA Netw Open*. 2020;3(6):e204207. doi:10.1001/jamanetworkopen.2020.4207

eTable 1. Associations Between Nicotine Replacement Therapy, Year, and Socioeconomic Position
eTable 2. Associations Between e-Cigarette Use and Year of Survey and SEP (Housing Tenure) in England, 2014 to 2019
eTable 3. Associations Between e-Cigarette Use and SEP (Housing Tenure) Stratified by Year in England, 2014-2019
eFigure 1. EC and NRT Use Among Those Who Quit Smoking for More Than 1 Year as a Percentage of Respective Use in the General Population
eFigure 2. EC Use Among Those Who Quit Smoking in the Past Year and Did not Use ECs in Quit Attempt as a Percentage of Respective Use in the General Population
eFigure 3. Current e-Cigarette Use Among Those Who Quit Smoking Before 2011 as a Percentage of Use in the General Population

This supplementary material has been provided by the authors to give readers additional information about their work.

© 2020 Kock L et al. *JAMA Network Open.*

Appendix

eTable 1. Associations Between Nicotine Replacement Therapy, Year, and Socioeconomic Position

eTable 1a. Associations between NRT use and year of survey and SEP among i) long-term ex-smokers and past-year ex-smokers (2014-2019).

	**Long term ex-smokers[a]**	***P***	**PY ex-smokers[b]**	***P***
	(N=19842)		(N=870)	
**SEP**				
ABC1	1 [reference]		1 [reference]	
C2DE	1.20 (0.81–1.79)	.36	2.87 (0.81-13.46)	.13
**Year**				
2014 ref	1 [reference]		1 [reference]	
2015	0.77 (0.52-1.14)	.19	0.54 (0.07-3.38)	.48
2016	0.90 (0.62-1.30)	.55	1.22 (0.22-6.90)	.86
2017	0.98 (0.69-1.41)	.92	1.22 (1.26-6.43)	.83
2018	0.91 (0.63-1.31)	.60	1.45 (0.31-7.63)	.66
2019	0.87 (0.58-1.29)	.43	2.52 (0.63-12.43)	.19
**Interaction terms**				
2015*C2DE	1.39 (0.80-2.43)	.25	0.94 (0.10-9.70)	.97
2016*C2DE	0.97 (0.55-1.70)	.92	0.09 (0.01-1.10)	.09
2017*C2DE	0.89 (0.51-1.55)	.68	0.30 (0.03-2.21)	.26
2018*C2DE	0.76 (0.43-1.36)	.36	0.20 (0.02-1.63)	.15
2019*C2DE	0.73 (0.37-1.40)	.46	1.50 (0.06-3.18)	.49

Ns are not weighted. All models are adjusted for age, sex and region. Results for prevalence of e-cigarette use are presented as Odds Ratios (95% CI) against the indicated referent. p<0.005 p values are indicated in bold. *p<0.01, **p<0.001. [a]Long-term (>1-year) ex-smokers; [b]Past year ex-smokers who did not use an NRT in their most recent quit attempt

SEP = Socio-economic position; ABC1 = higher SEP (higher and intermediate managerial, administrative and professional, supervisory occupations, clerical and junior managerial occupations); C2DE = lower SEP (semi-skilled and unskilled manual workers, state pensioners, casual and lowest grade workers, unemployed with state benefits only)

© 2020 Kock L et al. *JAMA Network Open.*

Table 1b: Associations between NRT use and SEP stratified by year among i) long-term ex-smokers and ii) past-year ex-smokers in England (2014-2019).

Year	2014	*P*	2015	*P*	2016	*P*	2017	*P*	2018	*P*	2019	*P*
**Long term ex-smokers[a]**	(N=3170)		(N=3462)		(N=3533)		(N=3617)		(N=3532)		(N=2528)	
**SEP**												
ABC1 (N=12008)	1 [reference]		1 [reference]		1 [reference]		1 [reference]		1 [reference]		1 [reference]	
C2DE (N=7834)	1.12 0.74-1.66)	.59	1.66 (1.11-2.48)	.02	1.22 (0.81-1.84)	.33	1.05 (0.71-1.55)	.80	0.89 (0.57-1.36)	.59	0.85 (0.49-1.45)	.60
**PY-ex-smokers[b]**	(N=194)		(N=158)		(N=129)		(N=152)		(N=152)		(N=85)	
**SEP**												
ABC1 (N=479)	1 [reference]		1 [reference]		1 [reference]		1 [reference]		1 [reference]		1 [reference]	
C2DE(N=391)	3.04 (0.85-14.34)	.11	3.16 (0.58-23.74)	.20	0.27 (0.01-2.23)	.27	1.10 (0.21-5.29)	.90	0.45 (0.06-2.50)	.38	1.26 (0.30-4.79)	.61

Ns are not weighted. All models are adjusted for age, sex and region. Results for prevalence of e-cigarette use are presented as Odds Ratios (95% CI) against the indicated referent. p<0.005 p values are indicated in bold. *p<0.01, **p<0.001.

[a]Long-term (>1-year) ex-smokers. [b]Past year ex-smokers who did not use an e-cigarette in their most recent quit attempt.

SEP = Socio-economic position; ABC1 = higher SEP (higher and intermediate managerial, administrative and professional, supervisory occupations, clerical and junior managerial occupations); C2DE = lower SEP (semi-skilled and unskilled manual workers, state pensioners, casual and lowest grade workers, unemployed with state benefits only)

© 2020 Kock L et al. *JAMA Network Open.*

eTable 2. Associations Between e-Cigarette Use and Year of Survey and SEP (housing tenure) in England, 2014 to 2019

	**Long term ex-smokers**	***P***	**PY ex-smokers**[b]	***P***	**Pre-2011 LT ex-smokers**[c]	***P***
	(N=19842)		(N=870)		(N=15063)	
**SEP**						
Other	1 [reference]		1 [reference]		1 [reference]	
Social housing	2.25 (1.39-3.55)	<.001	1.89 (0.55- 5.79)	.28	1.21 (0.28 - 3.73)	.77
**Year**						
2014 ref	1 [reference]	.01	1 [reference]		1 [reference]	
2015	1.46 1.08-2.00)	<.001	1.15 (0.44-2.93)	.79	0.54 (0.23 - 1.22)	.15
2016	2.37 (1.79-3.17)	<.001	1.37 (0.52-3.51)	.55	1.62 (0.85 - 3.17)	.15
2017	2.99 (2.27-3.97)	<.001	0.43 (0.11-1.30)	.15	1.51 (0.78 - 3.01)	.23
2018	3.25 (2.47-4.32)	<.001	1.16 (0.44-2.96)	.76	2.01 (1.07-3.90)	.03
2019	3.54 (2.67-4.75)	<.001	0.39 (0.06-1.54)	.42	3.27 (1.76 - 6.35)	<.001
**Interaction terms**						
2015*C2DE	0.90 (0.50-1.66)	.74	1.18 (0.20-6.72)	.88	2.48 (0.44-15.38)	.30
2016*C2DE	0.57 (0.31-1.05)	.07	0.19 (0.01-1.66)	.19	0.51 (0.06-3.50)	.51
2017*C2DE	**0.55 (0.31-0.98)**	.04	1.85 (0.25-13.31)	.53	1.67 (0.39-8.87)	.50
2018*C2DE	0.66 (0.54-1.40)	.14	0.21 (0.01-1.88)	.21	1.17 (0.26-6.26)	.83
2019*C2DE	0.79 (0.45-1.43)	.46	1.19 (0.04-18.01)	.65	0.92 (0.17-5.28)	.95

Ns are not weighted. All models are adjusted for age, sex and region. Results for prevalence of e-cigarette use are presented as Odds Ratios (95% CI) against the indicated referent. p<0.005 p values are indicated in bold. *p<0.01, **p<0.001.

[a]Long-term (>1-year) ex-smokers. [b]Past year ex-smokers who did not use an e-cigarette in their most recent quit attempt; [c]Long-term ex-smokers who quit smoking before 2011.

SEP = Socio-economic position; Other = higher SEP (mortgage bought, owned outright, private renting and other); Social housing = lower SEP ((local authority or housing association)

eTable 3. Associations Between e-Cigarette Use and SEP (Housing Tenure) Stratified by Year in England, 2014-2019

Year	2014	*P*	2015	*P*	2016	*P*	2017	*P*	2018	*P*	2019	*P*
**Long term ex-smokers[a]**	(N=3170)		(N=3462)		(N=3533)		(N=3617)		(N=3532)		(N=2528)	
**SEP**												
Other (N=12008)	1 [reference]		1 [reference]		1 [reference]		1 [reference]		1 [reference]		1 [reference]	
Social housing (N=7834)	2.31 (1.41-3.68)	<.001	1.99 (1.34-2.93)	<.001	1.27 (0.84-1.85)	.24	1.24 (0.88 - 1.71)	.21	1.46 (1.06 – 2.00)	.02	1.84 (1.28 - 2.59)	<.001
**PY-ex-smokers[b]**	(N=194)		(N=158)		(N=129)		(N=152)		(N=152)		(N=85)	
**SEP**												
Other (N=479)	1 [reference]		1 [reference]		1 [reference]		1 [reference]		1 [reference]		1 [reference]	
Social housing (N=391)	1.87 (0.55-5.68)	.30	(0.54-7.65)	.23	(0.02-2.05)	.33	(0.54-16.28)	.18	0.79 (0.04 – 5.24)	.84	3.07 (0.13 - 37.83)	.87
**Pre-2011 LT ex-smokers[c]**	(N=2683)		(N=2805)		(N=2703)		(N=2649)		(N=2501)		(N=1722)	
**SEP**												
Other (N=13105)	1 [reference]		1 [reference]		1 [reference]		1 [reference]		1 [reference]		1 [reference]	
Social housing (N=1958)	1.11 (0.25-3.42)	.87	2.76 (0.73 - 8.67)	.10	0.58 (0.09 - 1.98)	.47	1.84 (0.71 - 4.24)	.20	1.64 (0.60-3.77)	.28	1.16 (0.34-3.04)	.72

Ns are not weighted. All models are adjusted for age, sex and region. Results for prevalence of e-cigarette use are presented as Odds Ratios (95% CI) against the indicated referent. p<0.005 p values are indicated in bold. *p<0.01, **p<0.001.

[a]Long-term (>1-year) ex-smokers. [b]Past year ex-smokers who did not use an e-cigarette in their most recent quit attempt; [c]Long-term ex-smokers who quit smoking before 2011.

SEP = Socio-economic position; Other = higher SEP (mortgage bought, owned outright, private renting and other); Social housing = lower SEP ((local authority or housing association

© 2020 Kock L et al. *JAMA Network Open.*

eFigure 1. EC and NRT Use Among Those Who Quit Smoking for More Than 1 Year as a Percentage of Respective Use in the General Population

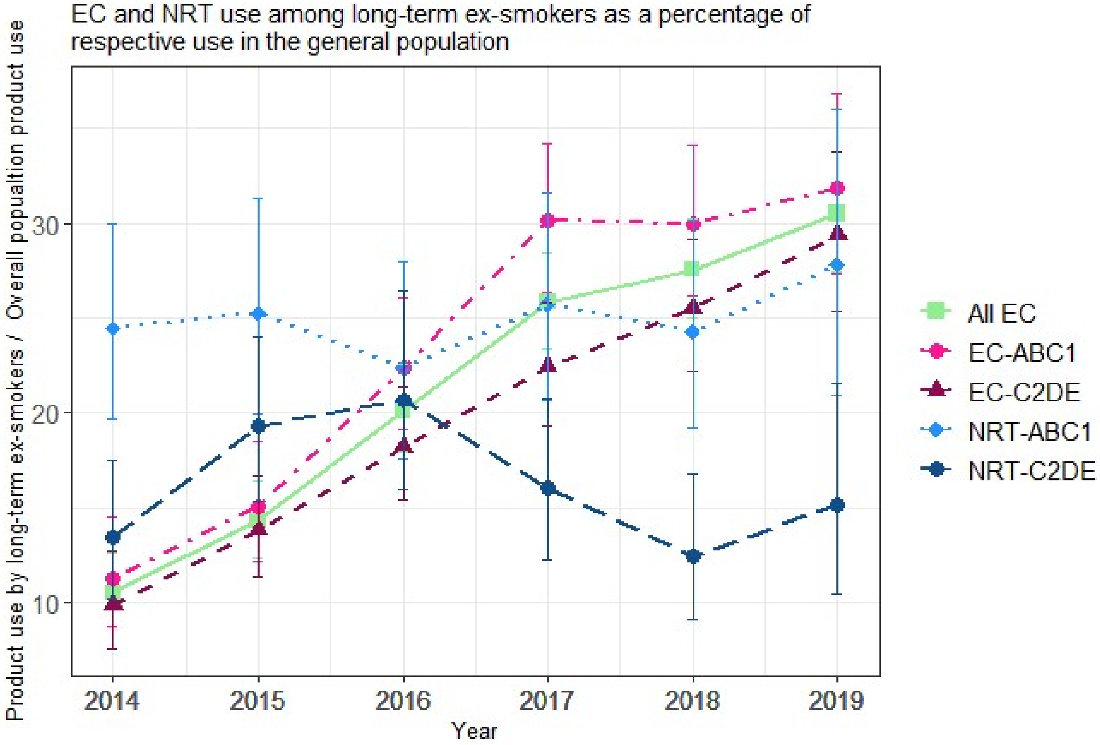

Ns are weighted

© 2020 Kock L et al. *JAMA Network Open.*

eFigure 2. EC Use Among Those Who Quit Smoking in the Past Year and Did not Use ECs in Quit Attempt as a Percentage of Respective Use in the General Population

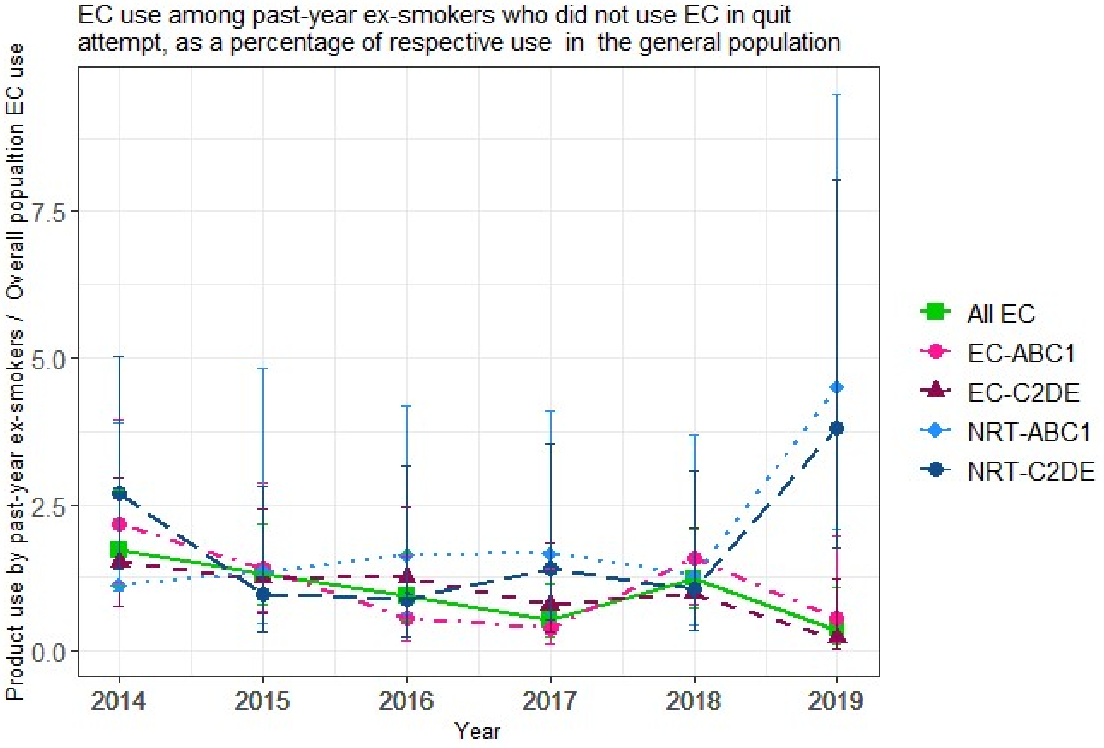

Ns are weighted

© 2020 Kock L et al. *JAMA Network Open.*

eFigure 3. Current e-Cigarette Use Among Those Who Quit Smoking Before 2011 as a Percentage of Use in the General Population

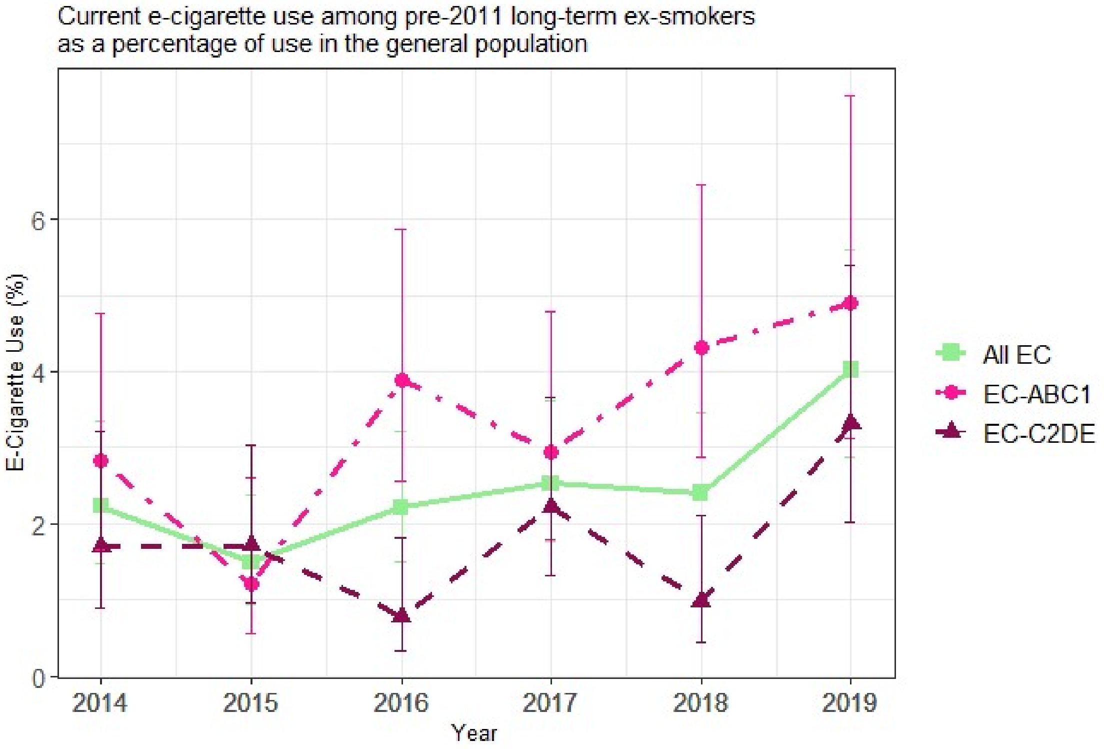

Ns are weighted

© 2020 Kock L et al. *JAMA Network Open.*
